# Effect of Treatment on Body Fluid in Patients with Unilateral Aldosterone Producing Adenoma: Adrenalectomy versus Spironolactone

**DOI:** 10.1038/srep15297

**Published:** 2015-10-19

**Authors:** Che-Hsiung Wu, Ya-Wen Yang, Szu-Chun Hung, Yao-Chou Tsai, Ya-Hui Hu, Yen-Hung Lin, Tzong-Shinn Chu, Kwan-Dun Wu, Vin-Cent Wu

**Affiliations:** 1 Division of Nephrology, Taipei Tzu Chi Hospital, Buddhist Tzu Chi Medical Foundation, Taipei, Taiwan; 2 School of Medicine, Tzu Chi University, Hualien, Taiwan; 3 Departments of Internal Medicine, National Taiwan University Hospital; 4 Division of General Surgery, Department of Surgery, National Taiwan University Hospital; 5 Division of Urology, Taipei Tzu Chi Hospital, Buddhist Tzu Chi Medical Foundation, Taipei, Taiwan; 6 Division of Endocrine and Metabolism, Taipei Tzu Chi Hospital, Buddhist Tzu Chi Medical Foundation, Taipei, Taiwan

## Abstract

Aldosterone affects fluid retention in the body by affecting how much salt and water that the kidney retains or excretes. There is limited information about the effect of prolonged aldosterone excess and treatment on body fluid in primary aldosteronism (PA) patients. In this study, body composition changes of 41 PA patients with unilateral aldosterone producing adenoma (APA) were assessed by a bio-impedance spectroscopy device. Patients with APA receiving adrenalectomy, as compared with those treated with spironolactone, had significantly lower relative overhydration (OH) and urine albumin excretion, and significantly higher urine sodium excretion four weeks after treatment. These differences dissipated 12 weeks after the initial treatment. Independent factors to predict decreased relative OH four weeks after treatment were male patients and patients who experienced adrenalectomy. Patients who underwent adrenaelctomy had significantly decreased TNF-α and increased serum potassium level when compared to patients treated with spironolactone 4 and 12 weeks after treatment. In this pilot study, we found that adrenalectomy leads to an earlier increase in renal sodium excretion and decreases in body fluid content, TNF-α, and urine albumin excretion. Adrenalectomy yields a therapeutic effect more rapidly, which has been shown to ameliorate overhydration in PA patients.

Primary aldosteronism (PA), characterized by inappropriate production of aldosterone, affects 5–13% of patients with hypertension^1^. Mineralocorticoid receptor (MR) antagonists (spironolactone) usually bring about target blood pressure (BP) control in patients with bilateral adrenal hyperplasia (BAH)^2^ with life-long medication. Aldosterone producing adenoma (APA) is the most common subtype that can be cured by adrenalectomy. The results of short-term, follow-up studies showed that unilateral adrenalectomy or treatment with MR antagonists is effective in correcting hypertension and hypokalemia in PA patients. However, normalization of blood pressure and correction of hypokalemia are not the only goals in managing PA. The excessive production of aldosterone in PA patients may lead to a higher rate of cardiovascular events and renal damage disproportionate to BP levels^3^,^4^.

Aldosterone affects fluid retention in the body by way of salt and water that the kidney retains or excretes. In PA, there is an increase in plasma volume due to increased sodium reabsorption^5^. A previous study demonstrated that patients with PA had greater values for plasma volume and extracellular fluid volume than did patients with essential hypertension^6^. There is limited information about the effect of prolonged aldosterone excess and treatment on body fluid. The clinical assessment of fluid status is relatively difficult because physical signs of edema are of limited value in diagnosing excess intravascular volume^7^. Bioimpedance spectroscopy is a simple and effective approach for the assessment of fluid status and for evaluating fat mass and lean mass^8^,^9^. The accuracy of fluid status and body composition measured by spectroscopy has been validated against available gold standard reference methods^10^,^11^. The main objective of this work was to compare the effect of adrenalectomy and spironolactone on body composition. Therefore, we performed a prospective study to evaluate the body composition changes as assessed by bio-impedance spectroscopy device in patients with APA treated with either adrenalectomy or spironolactone. We also assessed the changes of cardiac dysfunction, fluid overload, and renal function by novel biomarkers, and of the levels urine sodium and potassium excretion before and after treatment.

## Patients and methods

### Ethics Statement

The study complied with the Declaration of Helsinki and was approved by the institutional review board of National Taiwan University Hospital (Taipei, Taiwan) in addition to obtaining informed consent. All participants signed the informed consent form before inclusion in the study.

### Patient Selection

**Figure 1:**
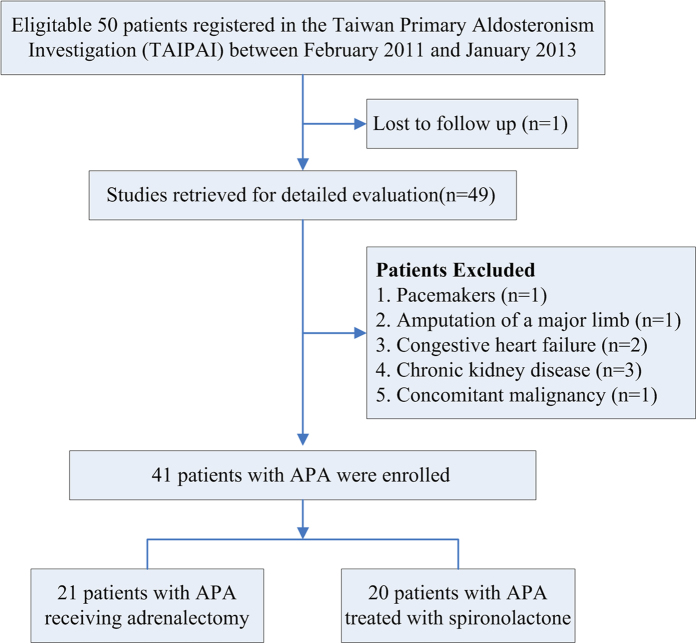
Trial profile. *Abbreviations: APA, aldosterone producing adenoma.

All patients were registered in the Taiwan Primary Aldosteronism Investigation (TAIPAI) between February 2011 and January 2013. This prospective study enrolled 21 patients diagnosed as APA and received adrenalectomy. Another 20 patients with APA treated with spironolactone were enrolled as a control group. It was the patient’s choice to receive adrenalectomy or medical therapy. The database was constructed for quality assurance at two medical centers and their four affiliated hospitals in different cities in Taiwan. Before confirmatory tests, all antihypertensive medications were discontinued for at least 21 days. Diltiazem and/or doxazosin were administered to control markedly high blood pressure when required^12^. The study excluded patients who had pacemakers/implanted defibrillators or amputation of a major limb, an acute disease, an obvious body weight change before enrollment (>5 kg in 3 months), pregnancy, or use of oral contraceptives. Additional exclusion criteria were clinically significant comorbid conditions such as uncontrolled hypertension, New York Heart Classification (NYHC) III-IV congestive heart failure, stage 3 or worse chronic kidney disease ([GFR] <60 mL/[min · 1.73 m^2^], or diagnosed with cancer within the previous 5 years (Fig. 1). We suggested a low salt diet with sodium intake less than 3,000 mg per day to all patients beginning 21 days before treatment and continuing throughout the study period. Medications that might interfere with the renin-aldosterone axis, including steroids, sex hormones, licorice, or non-steroidal anti-inflammatory drugs were also withheld. No patient took mineralocorticoid receptor antagonists (MRA) prior to participation in the study.

### PA Confirmation

Patients with an abnormal aldosterone-renin ratio (ARR) were confirmed to have PA by saline infusion tests and subsequent imaging studies for subtype identification (Figure S1). The diagnosis of APA was established in hypertensive patients with elevated ARR, TAIPAI score more than 60% and evidence for lateralized disease by adrenal CT (n = 41), NP59 scintigraphy (n = 13) or AVS (n = 22) (Text S1 and Figure S1).

### Study Protocol

All patients underwent measurements of BCM, blood pressure (BP), plasma N-terminal pro-brain natriuretic peptide (NT-proBNP), C reactive protein (CRP), tumor necrosis factor (TNF)-α, Cystatin C, and biochemistry before starting spironolactone (50 mg daily) treatments or receiving adrenalectomy. For patients treated with spironolactone, measurements were repeated after 4 and 12 weeks of medication. Patients who underwent unilateral adrenalectomy for adenoma took the second and third measurement at 4 and 12 weeks after the operation.

Blood pressure determinations were taken in the right arm after being seated for 5 min by a trained research assistant with a mercury sphygmomanometer with brachial cuff adjusted to the patient’s brachial circumference. The blood pressure was measured thrice at 5-minute intervals. The average value was used for analysis.

### BCM Measurements

Measurements of the fluid status were performed by a single well-trained nurse, using a portable whole-body bioimpedance spectroscopy device, the BCM (Fresenius Medical Care, Bad Homburg, Germany). The BCM measures the electrical responses at 50 different frequencies between 5 and 1000 kHz. Electrodes were attached to the hand and foot on the non-dominant side of the body after the patient had been in recumbent position for at least 5 minutes. Only one BCM measurement was performed for each individual patient because this method had good interobserver and intraobserver reproducibility^10^.

Fluid status assessed by the BCM is represented by overhydration (OH), which was derived from the impedance data based on a three compartment model^13^. The three compartments are lean tissue mass, adipose tissue mass, and OH. OH is the difference between the amount of extracellular water (ECW) in the tissue actually detected by the BCM and the amount of water present in tissue predicted using physiological models under normal (euvolemic) conditions. OH index defined as OH normalized to ECW and expressed as a percentage (%). All values of OH were compared with those obtained from an age and sex-matched healthy population. Fluid overload was defined as a relative OH index ≥7%, corresponding to the 90th percentile value for the reference cohort^14^. Relative lean tissue mass (LTM) and relative fat mass were presented as a percentage.

### Laboratory Measurements

The concentration of aldosterone was measured by radioimmunoassays with commercial kits (Aldosterone Maia Kit, Adaltis Italia S.p.A., Bologna, Italy). The lowest detectable concentration of aldosterone is 10.0 pg/mL. The normal range of aldosterone is 70–350 pg/mL in the upright position. PRA was measured as the generation of angiotensin I *in vitro* using a commercially available radioimmunoassay kit (Stillwater, MN, USA). The normal range of PRA was 2.63 ± 1.32 ng/mL/ h in the upright position. The mean (standard deviation [SD]) intra and interassay coefficients of variation (CVs) for the PRA assay were 1.9 (5.0%) and 4.5 (5.2%) respectively.

The plasma levels of TNF-α, CRP (R&D Systems, Minneapolis, MN) and NT-proBNP (USCN Life Science, Wuhan, P.R. China) were measured using the commercially available enzyme-linked immunosorbent assay kits according to the manufacturer’s instructions. Urine albumin excretion was defined as the urinary albumin-to-creatinine ratio (mg/mg) determined using the first morning void. Cystatin C was measured using a particle-enhanced immunonephelometric assay (N Latex Cystatin C; Siemens, Berlin, Germany) with a nephelometer (BNII; Siemens).

### Statistical Analysis

The data were presented as the mean values ± standard deviation. We used a logistic regression analysis to explore the factors for early decreased OH. The Mann-Whitney U test was used to determine the difference between the two treatment groups. The study assessed univariate correlations between relative OH and potential explanatory variables using Pearson’s correlation analyses.

To examine the effect of different treatment strategies on various time-sequential variables, we fitted the marginal linear regression models to the repeatedly measured responses using the generalized estimating equations (GEE) method^15^. We then calculated standardized regression coefficients and their 95% confidence intervals (CIs). GEE is efficient in achieving higher power with smaller sample size or lower number of repeated measurements in both complete and missing data scenarios^16^,^17^. We performed statistical analyses with SPSS for Windows, version 18.0 (SPSS Inc., Chicago, IL, U.S.A.) and R software, version 2.8.1 (Free Software Foundation, Inc., Boston, MA, U.S.A.). A normal distribution was attained by appropriate transformations of skewed variables such as aldosterone and ARR. The p-values <0.05 were considered statistically significant.

## Result

### Patient Characteristics

Twenty-one patients with APA receiving adrenalectomy (men, 12 (57.1%); age, 58.25 ± 21.92 years) and 20 patients with APA treated with spironolactone (men, 11 (55.0%); age, 57.25 ± 4.95 years) completed the BCM measurement and laboratory evaluation. Table 1 summarizes baseline subject characteristics before treatment. The operative group had significantly lower serum potassium and higher TNF-α level than those treated with spironolactone at enrollment. Baseline BCM profile was similar between patients in the two study groups.

### Factors Associated with Relative OH at Enrollment

**Figure 2:**
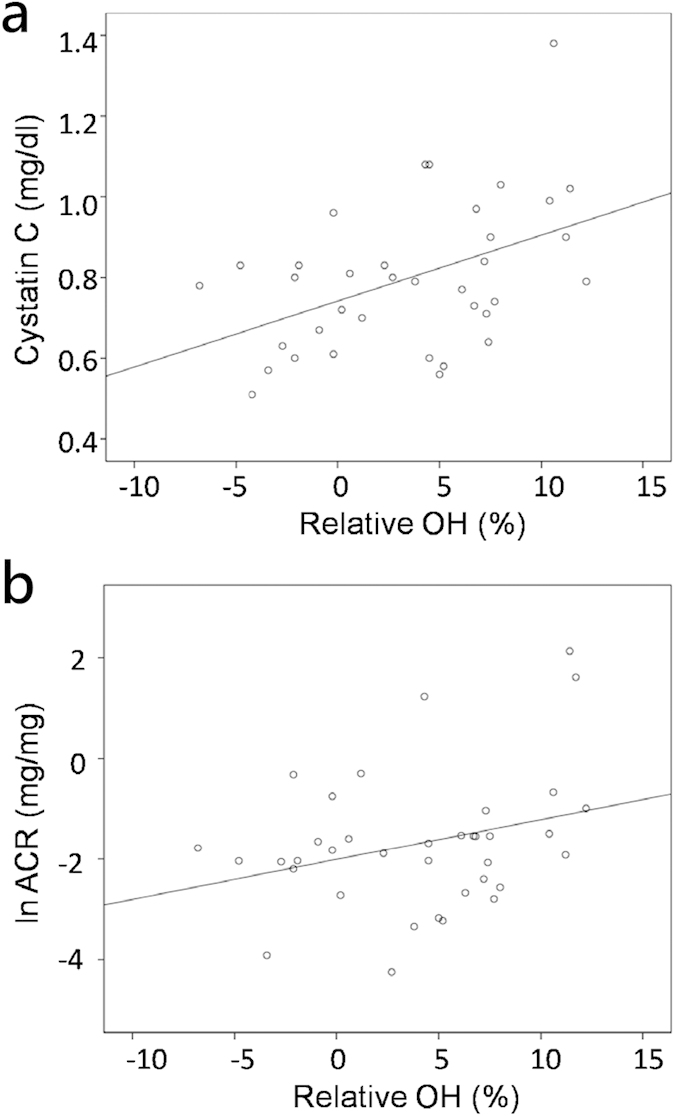
Factors associated with relative overhydration (OH) at baseline. Univariate analysis of correlations of baseline relative OH with the (**a**) cystatin C, (**b**) ln urine albumin-to-creatinine ratio (ACR)(**a**) cystatin C vs. relative OH at baseline r = 0.456, p = 0.005. (**b**) ln urine albumin-to–creatinine ratio (ACR) vs. relative OH at baseline r = 0.336, p = 0.042.

Correlations between baseline relative OH and other variables in the overall sample were assessed. Baseline relative OH was positively correlated with baseline serum Cystatin C (r = 0.456, p = 0.005) and urine albumin excretion (r = 0.336, p = 0.042) (Fig. 2).

### Time Dependent Variables after Treatment

**Figure 3:**
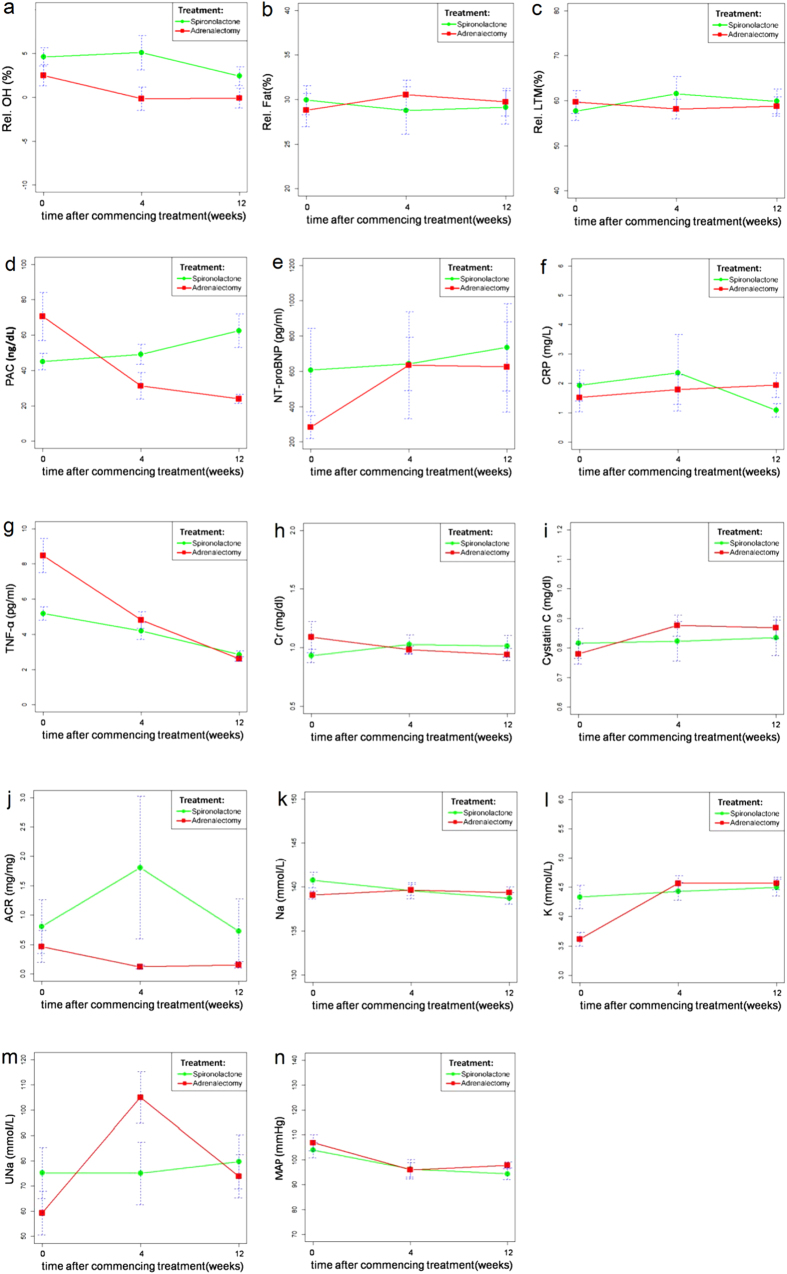
Generalized estimating equations to examine the effect of different treatment strategies on various time-sequential variables. Panels **a-c**: BCM parameters; Panels **d-g**: hormones and biomarkers; and Panels **h-n**: renal parameters. (**a**) The Relative overhydration (Rel. OH) between group, p = 0.062. Changes in time period contributed to changes in Rel. OH after 4 weeks (p = 0.011). Rel. OH was significantly different at 12 weeks (p = 0.008). (**b**) The Relative fat (Rel. Fat) between group, p = 0.034. Changes in time period contributed to changes in Rel. Fat after 4 weeks (p = 0.0105). (**c**) The Relative lean tissue mass (Rel. LTM) between group, p = 0.73. (**d**) The plasma aldosterone concentration (PAC) between group, p = 0.1942. PAC was significantly different at 4 weeks (p = 0.0034) and 12 weeks (p = 0.001). (**e**) The N-terminal pro-brain natriuretic peptide (NT-proBNP) between group, p = 0.448. (**f**) The C-reactive protein (CRP) between groups, p = 0.89. (**g**) The Tumor necrosis factor-alpha (TNF-α) between groups, p < 0.001. Changes in time period contributed to changes in TNF-α after 4 weeks (p < 0.001). TNF-α was significantly different at 12 weeks (p < 0.001). Changes in time period contributed to changes in TNF-α after 12 weeks (p < 0.001). (**h**) The serum creatinine (Cr) between groups, p = 0.46. (**i**)The cystatin C between groups, p = 0.570. (**j**) The urine albumin-to-creatinine ratio (ACR) between groups, p = 0.607. Changes in time period contributed to changes in ACR after 4 weeks (p = 0.003). (**k**) The serum sodium (Na) between groups, p = 0.49. (**l**) The serum potassium (K) between groups, p < 0.001. Changes in time period contributed to changes in K after 4 weeks (p < 0.001). Changes in time period contributed to changes in K after 12 weeks (p < 0.001). (**m**) The urine sodium (UNa) between groups, p = 0.26674. Changes in time period contributed to changes in UNa after 4 weeks (p < 0.001). (**n**) The mean arterial blood pressure (MAP) between groups, p = 0.7761. MAP was significantly different at 4 weeks (p = 0.003) and 12 weeks (p < 0.001).

Compared with the data at enrollment, relative LTM, serum creatinine, Cystatin C, sodium and CRP did not significantly change during the study period. Plasma aldosterone concentration (PAC) in operative group decreased over time and that of spironolactone group increased over time. In both groups, relative OH, TNF-α, and MAP (mean arterial blood pressure) showed a time-dependent decrease (p = 0.008, <0.001, and <0.001, respectively) after treatment (Fig. 3).

Using GEE model, relative OH, relative fat, urine sodium excretion, and urine albumin excretion change over time differed significantly between patients in the two study groups at four weeks after treatment. Patients in operative group, as compared with those in spironolactone group, had significantly lower relative OH (p = 0.011), urine albumin excretion (p = 0.003), and significantly higher urine sodium excretion (p < 0.001), relative fat (p = 0.034). Nonetheless, these differences dissipated at 12 weeks after the commencing treatment. Relative OH at 12 weeks was significantly decreased in both modalities as compared with that at baseline. A significant difference of serum potassium was observed between the two groups, especially the augmentation from baseline to 12 weeks (p < 0.001) in operative group. TNF-α attenuation over time differed significantly between patients in the two study groups at 4 (p < 0.001) and 12 weeks (p < 0.001) after treatment. Patients in operative group had significantly lower TNF-α than spironolactone group after treatment.

### Factor Predicted to Improve Relative OH after One Month

A larger proportion of patients in adrenalectomized group had decreased relative OH (p = 0.003) at four weeks. However, these kinds of trends were not significantly different at 12 weeks (Table 2).

In multivariate regression models using demographics, aldosterone profile, BCM profile, and other variables listed in Table 1, patients who received adrenalectomy (OR 30.9, p = 0.036) and were males (OR 85.1, p = 0.044) could predict decreased relative OH at four weeks after treatment.

## Discussion

To our knowledge, this is the first study to determine the body composition change after treatment in APA patients. We found that patients receiving adrenalectomy, as compared with those treated with spironolactone, had significantly lower relative OH, decreased TNF-α, urine albumin excretion, and higher urine sodium excretion at four week after treatment. Adrenalectomy might yield a therapeutic effect more rapidly, which has been shown to ameliorate cardiac hypertrophy in PA patients^18^.

### On Target Treatment of PA

Long-term exposure to high aldosterone levels, independent of blood pressure level, could eventually lead to cardiovascular and renal structural and functional damage^3^. Previous studies showed that cardiovascular and renal damages seem to benefit from treatment, yet the relative efficacy of adrenalectomy compared with spironolactone have not been clearly evaluated^19^,^20^. Our present study further suggests that relative hyperfiltration in PA improved soon after adrenalectomy as reflected by decreasing microalbuminuria. Factors for reversed hyperfiltration after treatment include decreased extracellular fluid volume and postoperative aldosterone suppression, both of which were found to occur earlier in adrenalectomized patients in the current study.

There are several concerns about the treatment with spironolactone. Findings regarding benefits of spironolactone use are limited partly due to frequent occurrence of dose-dependent side-effects, especially gynecomastia, among Asian patients^21^ and subsequent decrease in efficacy and medication compliance. As shown in an Italian study, 28% of PA patients discontinued MR antagonists treatment 22. Spironolactone treatment may also induce an increase in aldosterone, and subsequently hyperaldosterone trigger a vicious cycle with nongenomic effects that leads to an insufficient effect of prescribed spironolactone on blocking MR^21^. Another concern about spironolactone treatment is the slow onset of efficacy of spironolactone treatment, which is in agreement with our finding in this study. Regression of LV hypertrophy requires a longer time to occur in patients treated with spironolactone than in those treated with surgery, regardless of the level of BP changes^23^. As demonstrated by our previous prospective study, proteinuria dropped off soon after adrenalectomy but still failed to significantly decrease one year after initiation of spironolactone treatment^20^. Using data of the German population-based survey, Reincke *et al.* investigated the risk factors associated with morality in PA patients. As compared with medical treatment of PA, adrenalectomy was associated with reduced all-cause mortality and proved to be protective^24^. In a long-term follow-up study, Catena *et al.* found that both surgical and medical treatment of PA effectively reduce left ventricular mass. However, the effects of adrenalectomy occur earlier and are greater than those of spironolactone^25^. A prospective study investigating blood pressure control demonstrated that the adrenalectomized patients required significantly less drugs than medically treated^26^. Among Japanese patients with APA, amelioration of hypertension significantly associated with surgical treatment, while medical treatment showed no relationship^27^. This disparity might be explained by the differing duration, severity of disease, and spironolactone dose.

### Aldosteronism and Proinflammation

Most importantly, patients in the operative group had significantly attenuated TNF-α compared with patients in the spironolactone group. These results may be attributed directly to rapidly decreased PAC and possibly indirectly attributed to an earlier decrease in relative OH after adrenalectomy. Previous studies have demonstrated that PA is not merely an independent risk factor for CVD and cerebrovascular events^28^, but PA is also associated with increased level of oxidative stress and inflammatory biomarkers^29^,^30^. Aldosterone excess is detrimental not only through increased blood pressure but also through blood pressure independent pro-inflammatory effects on the heart and vessel walls^24^. A study has previously revealed that TNF-α mRNA and protein biosynthesis augmented in an adult feline myocardium resulted in volume overload^31^. The OH index was shown to be related to inflammation^32^ and a prediction to patients mortality^33^. Studies of Left Ventricular Dysfunction (SOLVD) have shown that circulating levels of TNF-α were elevated in patients with congestive heart failure, and that the level of TNF-α, leading to progressive cardiac dilatation and failure^34^, had a direct relationship with heart failure^35^. Adrenalectomy ameliorated aldosterone excess and led to an earlier improvement of volume overload, thus resulting in a more rapid decrease in TNF-α when compared to MR antagonist treatment.

### Aldosteronism and Fluid Overload

Our data showed volume overload, a process that might be contribute to high blood pressure, cardiovascular structural damage, and poor patient outcome, might be effectively ameliorated in patients who underwent adrenalectomy for APA during the study period. The increase in blood pressure and volume in PA patients caused glomerular hyperfiltration and microalbuminuria^20^,^36^. Sechi *et al.* showed that PA is characterized by partially reversible kidney dysfunction^37^ and that albuminuria is a marker of a hemodynamic rather than a structural renal defect. Renal insufficiency has been reported to occur in 7%^38^ to 29%^39^ and proteinuria in 8%^40^ to 24%^38^ of PA patients. Subtle kidney impairment may be masked by hyperfiltration before treatment and intrarenal hemodynamic adaptation to the effects of aldosterone excess^35^. However, after a prolonged period of exposure, hyperaldosteronism eventually results in loss of glomerluar function. Our study revealed that fluid overload might become evident in patients who had kidney function impairment. Serum Cystatin C, a marker of kidney function, and urine albumin excretion were positively correlated with baseline relative OH.

### Study Limitations

Our study has some limitations that should be acknowledged. We were unable to evaluate dietary fluid intake during the treatment period. Patients were over-hydrated if one’s sodium and water intake was excessive despite of treatment modality. Regarding medication treatment, we could not examine the dose-dependent effects of spironolactone. Furthermore, our study had a relatively small sample size. When there was no prior information on which to base a sample size, we were unable to present power analyses. However, the results of this pilot study provide evidence and guidance in the planning and design of further large scale trials.

## Conclusions

In this pilot study, we found that adrenalectomy leads to the normalization of plasma aldosterone concentration followed by an earlier increase in renal sodium excretion and decreases in overhydration, TNF-α, and urine albumin excretion than patients who received spironolactone. Though spironolactone achieves a similar therapeutic effect 12 weeks after treatment, the long-term effects of surgery and medication on cardiovascular and renal outcome remain unclear. Large-scale investigation of this finding will be necessary, and timely identification of PA is important to maximize the benefits of target treatment.

## Additional Information

**How to cite this article**: Wu, C.-H. *et al.* Effect of Treatment on Body Fluid in Patients with Unilateral Aldosterone Producing Adenoma: Adrenalectomy versus Spironolactone. *Sci. Rep.* **5**, 15297; doi: 10.1038/srep15297 (2015).

## Supplementary Material

Supplementary Information

## Figures and Tables

**Table 1:** Clinical and biochemical characteristics of study patients.

	Adrenalectomy	Spironolactone	p
Patients(n)	21	20	
**Demography**			
Male (%)	12(57.1%)	11(55.0%)	0.766
Age (y/o)	58.25 ± 21.92	57.25 ± 4.95	0.754
BMI(kg/m^2^)	24.9 ± 4.1	24.5 ± 3.1	0.897
MAP(mmHg)	107.7 ± 14.2	103.9 ± 14.2	0.421
DM, n (%)	2 (9.5%)	3 (15%)	0.643
**Aldosteronism profile**			
Sodium (mmol/L)	139.1 ± 1.9	140.8 ± 3.5	0.157
Potassium (mmol/L)	3.62 ± 0.52	4.34 ± 0.88	0.004
PAC (ng/dL) *	38.1(33.3–54.7)	53.3(36.5–80.7)	0.149
Log ARR	2.42 ± 0.68	1.92 ± 0.86	0.082
UNa (mmol/L)	59.3 ± 34.8	73.1 ± 44.5	0.347
UK (mmol/L)	31.6 ± 20.8	33.1 ± 13.6	0.443
**Body composition**			
Relative OH (%)	2.51 ± 5.45	5.12 ± 4.63	0.176
Relative fat (%)	28.8 ± 8.5	28.9 ± 6.9	0.897
Relative LTM (%)	59.7 ± 11.2	59.0 ± 8.8	0.988
**Other variables**			
HOMA-IR (mU/L·mmol/L)	39.4 ± 40.0	45.8 ± 62.7	0.664
C-reactive protein (mg/L)	1.52 ± 2.13	1.93 ± 2.16	0.433
TNF-α (pg/ml)	8.47 ± 4.23	5.18 ± 1.56	0.009
NT-proBNP (pg/ml)	2.83 ± 2.82	6.07 ± 9.78	0.165
ACR (mg/mg)	0.47 ± 0.12	0.81 ± 0.21	0.393
Creatinine (mg/dl)	1.09 ± 0.59	0.93 ± 0.26	0.574
Cystatin C (mg/dl)	0.78 ± 0.14	0.82 ± 0.22	0.779
**Categories of hypertensive drugs before recruitment**			
β- blockers (%)	7 (33.3)	5 (25)	0.789
ACEI/ ARB (%)	6 (28.6)	7 (35)	0.542

Abbreviations: ACEI, angiotensin-converting enzyme inhibitors; ACR, Urine albumin-to-creatinine ratio ; APA, aldosterone producing adenoma; ARB, angiotensin II receptor blockers; ARR, aldosterone-renin ratio (ng/dL per ng/mL/h); BMI, body mass index; DM, Diabetes mellitus; HOMA-IR, homeostasis model assessment for insulin resistance; MAP, mean arterial blood pressure; NT-proBNP, N-terminal pro-brain natriuretic peptide; LTM, lean total mass; OH, overhydration; PAC, plasma aldosterone concentration ; TNF-α, Tumor necrosis factor-alpha; UK, urine potassium; UNa, urine sodium.

Data were provided as the mean values ± standard deviation, Significance was determined by Wilcoxon signed rank test in nonparametric distribution.

*Median (interquartile range).

Note: To convert potassium in mmol/L to mEq/L, multiple by 1; PAC in ng/dL to nmol/L, multiple by 0.02774; PRA in ng/mL/hr to ng/(Lxs), multiple by 0.2778.

**Table 2:** Relative Risks and 95% Confidence Intervals of Independent Factors to predict decreased overhydration 4 weeks after treatment by Multivariate Logistic Regression Model.

Variable	β	Wald	*p*	Odds ratio	95% CI
Adrenalectomy	3.431	4.382	0.036	30.9	1.244 767.7
Male sex	4.444	4.072	0.044	85.1	1.136 6374

*All covariates listed in Table 1 are put into multivariate logistic regression model.
