# Supplementary material for: Effect of Treatment on Body Fluid in Patients with Unilateral Aldosterone Producing Adenoma: Adrenalectomy versus Spironolactone
Source: Sci Rep. 2015 Oct 19;5:15297. doi: 10.1038/srep15297 (PMC4609981; doi:10.1038/srep15297)
Supplement: Supplementary Information [file srep15297-s1.doc]

**Effect of Treatment on Body Fluid in Patients with Unilateral Aldosterone Producing Adenoma: Adrenalectomy versus Spironolactone**

Che-Hsiung Wu^1, 2^**,** Ya-Wen Yang^4^**,** Szu-Chun Hung^1, 2^**,** Yao-Chou Tsai^5^, Ya-Hui Hu^6^, Yen-Hung Lin^3^, Tzong-Shinn Chu^3^**,** Kwan-Dun Wu^3^, Vin-Cent Wu^3*^

^1^ Division of Nephrology, Taipei Tzu Chi Hospital, Buddhist Tzu Chi Medical Foundation, Taipei, Taiwan

^2^ School of Medicine, Tzu Chi University, Hualien, Taiwan

^3^ Departments of Internal Medicine, National Taiwan University Hospital

^4^ Division of General Surgery, Department of Surgery, National Taiwan University Hospital

^5^ Division of Urology, Taipei Tzu Chi Hospital, Buddhist Tzu Chi Medical Foundation, Taipei, Taiwan

^6^ Division of Endocrine and Metabolism, Taipei Tzu Chi Hospital, Buddhist Tzu Chi Medical Foundation, Taipei, Taiwan

Correspondence to:

Vin-Cent Wu, MD, PhD

Room 1419, Clinical Research Building, Department of Internal Medicine

National Taiwan University Hospital

7 Chung-Shan South Road, Taipei 100, Taiwan

Telephone: +886-2-23562082

Fax: +886-2-23934176

E-mail: q91421028@ntu.edu.tw

***Material and methods***

**Enrolled hospitals**

This study included two medical centers (National Taiwan University Hospital (NTUH), Taipei; Taipei University Hospital, Taipei), four metropolitan hospitals (Cardinal Tien Hospital, New Taipei City; Taipei Tzu Chi Hospital, New Taipei City; Yun- Lin Branch of NTUH, Douliou City; Tao-Yuan Hospital, Taoyuan City), and two local hospitals (Hsin-Chu Branch of NTUH, Hsin-Chu City; Zhongxing Branch of Taipei City Hospital, Taipei).

**The standard protocol to identify aldosteronism**

The diagnosis of aldosteronism was established in hypertensive patients on the basis of the following criteria: (Fig S1)

***Confirmation***

Fulfillment of the following three conditions confirms a diagnosis of aldosteronism: (1) autonomous excess aldosterone production evidenced with an ARR > 35; (2) a TAIPAI score larger than 60%; (3) post-saline loading PAC > 10 ng/dl, or PAC/PRA> 35 (ng/dL)/ (ng/mL/h) shown in a post capotopril/ losartan test, or PAC>6 ng/dL indicated by a fludrocortisone suppression test. (Abbreviations: PAC, plasma aldosterone concentration; PRA, plasma renin activity)

***Lateralization***

1. APA is identified on the basis on the following four conditions: (1) autonomous excess aldosterone production evidenced with an ARR > 35, a TAIPAI score larger than 60%, and post-saline loading PAC > 10 ng/dl; (2) adenoma evidenced with a CT scan for pre-operative evaluation;[6] (3) lateralization of aldosterone secretion at AVS or during dexamethasone suppression NP-59 SPECT/CT; (4) pathologically proven adenoma after an adrenalectomy for those with operations, and subsequent emergence of either a cure pattern of hypertension without anti-hypertensive agents or improvement in hypertension, potassium, PAC, and PRA.
2. Idiopathic hyperaldosteronism (IHA) is distinguished on the basis on the following four criteria: (1) autonomous excess aldosterone production evidenced with an ARR > 35, a TAIPAI score larger than 60%, and post-saline loading PAC > 10 ng/dl (2) evidence of bilateral diffuse enlargement indicated by a CT scan for pre-operative evaluation; (3) non-lateralization of aldosterone secretion at AVS or during dexamethasone suppression NP-59 SPECT/CT; (4) evidence of diffuse cell hyperplasia reported in following pathology studies for those with operations.

**Figure S1**. The subtype-differentiating protocol of the TAIPAI group .


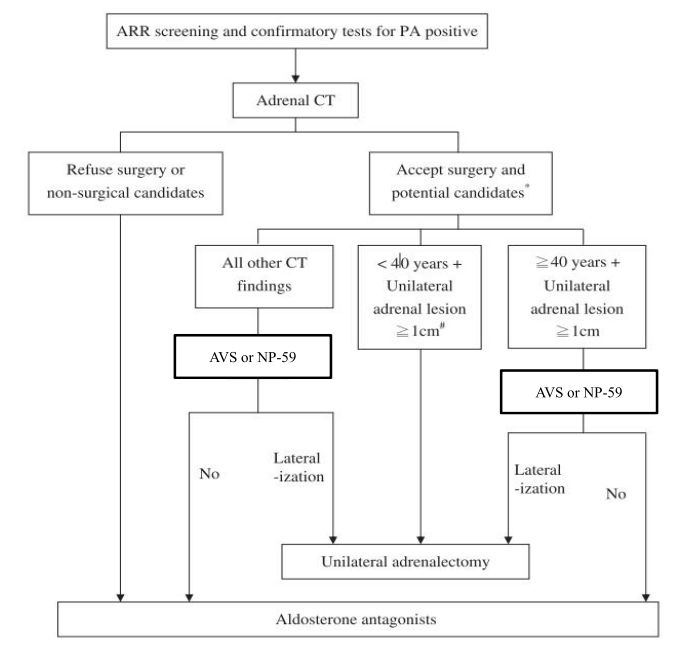


Abbreviations: AVS, adrenal venous sampling; APA, aldosterone-producing adenomas; ARR, aldosterone-to-renin ratio; CT, computed tomography; NP-59- SPECT, I-131-6-beta-iodomethyl-19-norcholesterol single-photon emission computed tomography.

Membership of the Taiwan Primary Aldosteronism Investigation (TAIPAI) Study Group: Jui-Hsiang Lin, MD(Tao-Yuan General Hospital, PI of Committee); Wei-Jie Wang, MD, PhD (Tao-Yuan General Hospital, PI of Committee); Che-Hsiung Wu, MD(Taipei Tzu Chi Hospital, PI of Committee); Vin-Cent Wu, MD(NTUH, PI of Committee); Yen-Hung Lin, MD(NTUH, PI of Committee); Yi-Luwn Ho, MD, PhD(NTUH, PI of Committee); Hung-Wei Chang, MD, PhD(Far eastern hospital, PI of Committee); Lian-Yu Lin MD, PhD(NTUH, PI of Committee); Fu-Chang Hu, MS, ScD, Harvard statitics, Site Investigator); Kao-Lang Liu, MD(NTUH, PI of Committee); Shuo-Meng Wang, MD(NTUH, PI of Committee); Kuo-How Huang, MD(NTUH, PI of Committee); Yung-Ming Chen, MD(Yun-Lin Branch, NTUH, PI of Committee); Chin-Chi Kuo, MD(Yun-Lin, PI of Committee); Chin-Chen Chang, MD(NTUH, PI of Committee); Shih-Cheng Liao, MD(NTUH, PI of Committee); Ruoh-Fang Yen, MD, PhD(NTUH, PI of Committee); Kwan-Dun Wu, MD, PhD(NTUH, Director of Coordinating Center).

1. Wu VC, Hu YH, Wu CH, Kao CC, Wang CY, et al. (2014) Administrative data on diagnosis and mineralocorticoid receptor antagonist prescription identified patients with primary aldosteronism in Taiwan. J Clin Epidemiol.

2. Wu VC, Lo SC, Chen YL, Huang PH, Tsai CT, et al. (2011) Endothelial Progenitor Cells in Primary Aldosteronism: A Biomarker of Severity for Aldosterone Vasculopathy and Prognosis. J Clin Endocrinol Metab 96: 3175-3183.

3. Sechi LA, Novello M, Lapenna R, Baroselli S, Nadalini E, et al. (2006) Long-term renal outcomes in patients with primary aldosteronism. Jama 295: 2638-2645.

4. Wu VC, Chang HW, Liu KL, Lin YH, Chueh SC, et al. (2009) Primary Aldosteronism: Diagnostic Accuracy of the Losartan and Captopril Tests. Am J Hypertens 22: 821-827.

5. Kuo CC, Wu VC, Huang KH, Wang SM, Chang CC, et al. (2011) Verification and evaluation of aldosteronism demographics in the Taiwan Primary Aldosteronism Investigation Group (TAIPAI Group). J Renin Angiotensin Aldosterone Syst 12: 348-357.

6. Chao CT, Wu VC, Kuo CC, Lin YH, Chang CC, et al. (2013) Diagnosis and management of primary aldosteronism: an updated review. Ann Med 45: 375-383.

7. Wu VC, Yang SY, Lin JW, Cheng BW, Kuo CC, et al. (2011) Kidney impairment in primary aldosteronism. Clin Chim Acta 412: 1319-1325.

8. Yen RF, Wu VC, Liu KL, Cheng MF, Wu YW, et al. (2009) 131I-6beta-iodomethyl-19-norcholesterol SPECT/CT for primary aldosteronism patients with inconclusive adrenal venous sampling and CT results. J Nucl Med 50: 1631-1637.

9. Wu VC, Chao CT, Kuo CC, Lin YH, Chueh SC, et al. (2012) Diagnosis and Management of Primary Aldosteronism. Acta Nephrologica 26: 111-120.
